# Editorial: Factors for the progression from latent tuberculosis infection to tuberculosis disease

**DOI:** 10.3389/fcimb.2023.1225259

**Published:** 2023-06-29

**Authors:** Cheng Chen, Leonardo Martinez, Biao Xu

**Affiliations:** ^1^ Department of Chronic Communicable Diseases, Jiangsu Provincial Center for Disease Control and Prevention, Nanjing, China; ^2^ Department of Epidemiology, School of Public Health, Boston University, Boston, MA, United States; ^3^ School of Public Health, Fudan University, Shanghai, China

**Keywords:** tuberculosis, latent tuberculosis infection - LTBI, diagnostics, biomarkers, IGRA

Nearly one-fifth of the world population was infected with *Mycobacterium* tuberculosis (Gao et al., 2015), and about 10% of the infected population will finally progress to tuberculosis (TB) disease. The risk of progressing to TB disease was significantly high in the first 2 years of infection (Reichler et al., 2018). However, current diagnostics can’t distinguish the infection between recent and remote. More importantly, there is no valid diagnostics at present that could predict the risk of progress to TB disease in people with latent tuberculosis infection (LTBI). Recently, human genetic, immunological and metabolic biomarkers have been considered having promising prospects in monitoring and predicting TB risks qualitatively and quantitatively (Scriba et al., 2021). LTBI screening in TB high-risk groups, such as people living with HIV (PLHIV), people receiving immunosuppressive treatment and the elders are also helpful for early detection of TB disease (Anonymous, 2021).

There are seven manuscripts under this thematic issue covering topics of cytokines/chemokines in TB progression, LTBI status among PLHIV, TB outbreaks, and LTBI testing among diabetics. Selecting appropriate tuberculin skin test (TST) cut-off values for students receiving tuberculosis preventive treatment (TPT) are critical in outbreak investigation and transmission prevention. Barriers for receiving TPT among health care workers (HCWs) are still observed in some circumstances. High risk to TB disease among the LTBI elderly population in high TB burden areas call for actions on TB screening.

The study “*Elevated IP-10 at the protein and gene level associates with pulmonary TB*” adopted multiplex ELISA method to quantify 27 circulatory markers present within the unstimulated plasma of healthy, LTBI and TB individuals. Interferon gamma inducible protein (IP-10) was found abundant among TB patients at protein and RNA level, suggesting IP-10 might be a potential biomarker for TB disease (Fisher et al.).

The study “*Positive rate and risk factors of latent tuberculosis infection among persons living with HIV in Jiangsu Province, China*” was a cross-sectional study which was carried out among PLHIV in Jiangsu Province from June to July 2021 (Zhang et al.). Considering the fact that LTBI testing has no “gold standard”, this study applied Tuberculin skin test (TST), ESAT6-CFP10 test (EC), and QuantiFERON-TB gold in-tube (QFT) to detect LTBI among PLHIV. Findings from this study show that QuantiFERON-TB gold in-tube (QFT) report the highest LTBI prevalence in PLHIV.

Chen et al. reported a cross-sectional study on “*The association between diabetes status and latent-TB interferon gamma release assay (IGRA) levels from a cross-sectional study in eastern China*”. LTBI screening was given to 5,302 people in eastern China. The QuantiFERON-TB Gold In-Tube (QFT) assay was performed in LTBI diagnosis. Fasting plasma glucose (FPG) was tested in each participant. The diagnosis of diabetes followed the guidelines of the American Diabetes Association. Participants were classified into normoglycemia, prediabetes, undiagnosed diabetes, and previously diagnosed diabetes. The relationship between the QFT TB antigen and diabetes status was analyzed. They found diabetes status had little influence on the level of QFT TB antigen response among IGRA-positive persons.

Xiao et al. reported an epidemiological study among students and the elders titled as “*Prevalence of latent tuberculosis infection and incidence of active tuberculosis in school close contacts in Shanghai, China: Baseline and follow-up results of a prospective cohort study*”. They established a cohort study composed by freshman/sophomore TB patients’ close contacts in three administrative districts in Shanghai, China, and completed a 2-year follow-up. The prevalence of LTBI was 4.8% measured by QFT. During the 2-year follow-up, three clinically confirmed cases of TB were detected. The 2-year cumulative incidence was 0.4% (95%CI 0.1-1.2) with a median duration of 1 year for TB occurrence. The incidence rate of active TB was 2.0 per 1000 person-years with a total of 1497.3 observation person-years. These results suggests school close contacts should be considered as key high risk group for LTBI testing and management. Another study in school students by Lu et al. was the “*Selection of the cutoff value of the tuberculin skin test for diagnosing students who need preventive treatment: A school-based cross-sectional study*”. They revealed that if there were three or more cases of students with tuberculosis in a class or if the moderate or strong TST positivity rate was much higher than the normal range in the region, attention should be paid to those with moderately positive TST results. Interferon-gamma release assays (IGRAs) are recommended for follow-up TSTs. Concerns were also given to the elderly population. In the study of “*The burden and predictors of latent tuberculosis infection among elder adults in high epidemic rural area of tuberculosis in Zhejiang, China*”, adults aged 65 years and older living in the rural areas in Zhejiang Province were screening twice in a 12-month duration between 2021 and 2022 to determine the TB incidence in this population (Wang et al.). A total of 1856 individuals enrolled for LTBI testing. One-third (33.4%) of participants had abnormal chest radiographs and 34.9% participants were diagnosed as LTBI. Nine participants (0.5%) developed active TB during this one-year follow-up period. People who frequently went to closed entertainment places such as chess and card playing rooms had a relatively high prevalence of LTBI (39.5%). This study showed that LTBI was prevalent among the elder adults living in TB high-epidemic rural areas in Zhejiang province. Elderly men, smokers, and people having poor ventilation at home were prone to high risk of LTBI, and regular LTBI screening should be considered to these people.

In general, TB preventive treatment (TPT) for LTBI was acceptable for HCWs. However, the study titled as “*Large gap between attitude and action in tuberculosis preventive treatment among tuberculosis-related healthcare workers in eastern China*” found that TB-associated HCWs in eastern China held different attitudes from positive to negative toward TPT, and there was a large gap between attitudes and actual action for getting the testing and accepting the treatment (Wang et al.).

## Author contributions

CC wrote the manuscript, BX and LM edited the manuscript. All authors contributed to the article and approved the submitted version.
